# Second Derivative Synchronous Fluorescence Spectroscopy for the Simultaneous Determination of Chlorzoxazone and Ibuprofen in Pharmaceutical Preparations and Biological Fluids

**Published:** 2009-06

**Authors:** N. El-Enany, F. Belal, Y. El-Shabrawy, M. Rizk

**Affiliations:** 1 *Department of Analytical Chemistry, Faculty of Pharmacy, University of Mansoura, Mansoura, Egypt;*; 2 *Faculty of Pharmacy and Health Science, Ajman University of Science and Technology, United Arab Emirates;*; 3 *Department of Analytical Chemistry, Faculty of Pharmacy, University of Helwan, Cairo, Egypt.*

**Keywords:** chlorzoxazone, ibuprofen, synchronous fluorimetry, pharmaceutical preparations and biological fluids

## Abstract

A rapid, simple and highly sensitive second derivative synchronous fluorometric method has been developed for the simultaneous analysis of binary mixture of chlorzoxazone (CLZ) and ibuprofen (IP). The method is based upon measurement of the synchronous fluorescence intensity of these drugs at Δλ=60 nm in methanol. The different experimental parameters affecting the fluorescence of the two drugs were carefully studied and optimized. The fluorescence-concentration plots were rectilinear over the range of 0.2-4 μg/mL and 0.1-1.6 μg/mL for CLZ and IP, respectively with lower detection limits (LOD) of 0.028 and 8.3 × 10^-3^ μg/mL and quantification limits (LOQ) of 0.086 and 0.03 μg/mL for CLZ and IP, respectively. The proposed method was successfully applied for the determination of the two compounds in synthetic mixtures and in commercial capsules. The high sensitivity attained by the proposed method allowed the determination of both drugs and real human plasma samples. The mean % recoveries in real human plasma (n=3) were 87.69 ± 6.15 and 92.57 ± 4.39 for each of CLZ and IP respectively.

## INTRODUCTION

**Figure 1:**
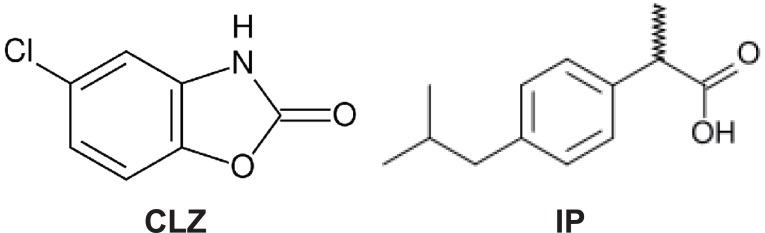
Structural formulae of the studied drugs.

Chlorzoxazone (CLZ), 5-chloro-2(3H)-benzoxazolone (Fig. 1) is a centrally acting muscle relaxant used to treat muscle spasms. It acts on the spinal cord by depressing reflexes (1).

A spectrofluorimetric method (2) has been reported for the determination of CLZ either *per se* or in pharmaceutical preparations and biological fluids. The United States Pharmacopoeia (USP) recommends spectrophotometric method for its determination in pure form (3) and HPLC method for its determination in tablets.

Ibuprofen (IP) is α-Methyl-4-(2-methylpropyl) benzene-acetic acid (Fig. 1). It is used for relief of symptoms of arthritis, primary dysmenorrhoea, fever, and as an analgesic, especially where there is an inflammatory component.

Regarding ibuprofen, several spectrofluorimetric methods (4-6) have been described for its analysis, whether in dosage forms or in biological fluids. British Pharmacopoeia recommended direct titration against sodium hydroxide using phenolphethaline as indicator for analysis of raw material (7), while USP recommends an HPLC technique for its analysis (7).

The normal synchronous fluorescence spectra of CLZ and IP greatly overlap. This observation led us to utilize second derivative synchronous fluorescence spectroscpoy (SDSFS) to solve this problem by measuring peak intensities at 306 and 230 nm for CLZ and IP respectively. The method developed was applied to the simultaneous determination of CLZ and IP in co-formulated pharmaceutical preparation.

Synchronous fluorescence spectroscopy (SFS) has several advantages over conventional fluorescence spectroscopy, including simple spectra, high selectivity and low interference (8). Because of its sharp, narrow spectrum, SFS serves as a very simple, effective method of obtaining data for quantitative determination in a single measurement (9).

The combination of SFS and derivatives is more advantageous than the conventional emission spectrum in terms of sensitivity, because the amplitude of the derivative signal is inversely proportional to the band width of the original spectrum (10, 11).

Recently, derivative synchronous fluorometry (DSF) has been utilized for determination of several mixtures in their co-formulated dosage forms and biological fluids. Thus mixtures of cinnarizine and domperidone (12), metoclopramide and pyridoxine (13), aspirin with salicylic acid (14), diflunisal and salicylic acid (15), carvedilol and ampicillin (16) have been determined through this approach.

To the best of our knowledge, neither conventional nor synchronous spectrofluorometry has been reported for the analysis of CLZ and IP in binary mixtures.

## EXPERIMENTAL

### Material

Chlorzoxazone and Ipuprofen pure samples were purchased from Sigma (St. Louis, Mo, USA) and used as received. Myofen capsules labeled to contain 250 mg of CLZ and 200 mg of IP in a ratio of 5:4 (Batch # 604346) was obtained from commercial source in the local market.

### Reagents

All reagents and solvents were of Analytical Reagent Grade.

- Methanol (Merck, Darmstadt, Germany). Acetate buffer 0.2 M (pH3.6-5.6) was prepared by mixing appropriate volume of 0.2 M acetic acid with 0.2 M sodium acetate. Borate buffers (pH6-9.5) were prepared by mixing appropriate volumes of 0.02 M boric acid with 0.2 M sodium hydroxide.
- Chloroform (Aldrich, St. Louis, MO, USA). Phosphate buffer (pH7.4) was prepared by mixing appropriate volume of 0.2 M KH_2_PO_4_ with 0.1 M sodium hydroxide (17).
- Sodium hydroxide ((BDH, UK), 0.1 M aqueous solution.

### Apparatus

Fluorescence spectra and measurements were recorded using a Perkin-Elmer UK model LS 45 luminescence spectrometer, equipped with a 150 Watt Xenon arc lamp, gratting excitation and emission monochromators for all measurements and a Perkin-Elmer recorder. Slit widths for both monochromators were set at 10 nm. A 1 cm quartz cell was used. Derivative spectra can be evaluated using Fluorescence Data Manger (FLDM) software.

For best resolution and smoothing, use number of points of 99 for deriving the second derivative spectra. The fluorescence intensities of the second derivative spectra were estimated at 306 nm and 230 nm for CLZ and IP, respectively.

A pH Meter (Model pHS-3C, Shanghai Leici instruments Factory, China) was used for pH adjustment.

### Standard Solutions

Stock solutions of CLZ and IP were prepared by dissolving 10.0 mg of the studied compounds in 100 mL of methanol and were further dilute with the same solvent as appropriate. The standard solutions were stable for 10 days when kept in the refrigerator.

## RECOMMENDED PROCEDURES

### Calibration curve

Aliquots of CLZ and IP standard solutions covering the working concentration range cited in Table 1 were transferred into a series of 10 mL volumetric flasks. Two mL of borate buffer (pH7.0) was added and the solutions were diluted to the volume with methanol and mixed well. Synchronous fluorescence spectra of the solutions were recorded by scanning both monochromators at a constant wavelength difference Δλ=60 nm and scan rate of 600 nm min^-1^ using 10 nm excitation and emission windows. The second derivative fluorescence spectra of CLZ and IP was derived from the normal synchronous spectra using FLDM software. The peak amplitude of the second derivative spectra was estimated at 306 nm and 230 nm for CLZ and IP, respectively. A blank experiment was performed simultaneously. The peak amplitude of the second derivative technique was plotted *vs* the final concentration of the drug (μg/mL) to get the calibration graph. Alternatively, the corresponding regression equations were derived.

### Procedure for the synthetic mixture

Aliquot volumes of CLZ and IP standard solutions in the ratio of 5:4 were transferred into a series of 10 mL volumetric flasks. Two mL of borate buffer (pH7.0) was added followed by dilution to the volume with methanol, and mixed well. The recommended procedure under calibration curve was then performed. The peak amplitude of the second derivative technique was plotted *vs* the final concentration of the drug (μg/mL) to generate the calibration graph. Alternatively, the corresponding regression equations were derived.

### Applications

**Procedure for commercial capsules.** The contents of 10 capsules were emptied and mixed well. A weighed quantity of the powder equivalent to 10.0 mg CLZ and 8.0 mg of IP (in their pharmaceutical ratio of 5:4) was transferred into a small conical flask and extracted with 3 × 30 mL of methanol. The extract was filtered into a 100 mL volumetric flask. The conical flask was washed with few mLs of methanol. The washings were passed into the same volumetric flask and completed to the mark with the same solvent. Aliquots covering the working concentration range were transferred into 10 mL volumetric flasks. The recommended procedure under "Calibration Curve" was performed. The nominal content of the capsules were determined either from a previously plotted calibration graph or using the corresponding regression equation.

**Procedure for real human plasma.** Myofen capsule (250 mg CLZ and 200 mg IP/capsule) was orally administered to healthy fasting volunteer (male, 40 years old). 5 mL of blood sample was withdrawn after two hours, 4 mLs of citrate solution were added, and centrifuged at 3500 rpm for 15 min. to obtain 3.0 mL of plasma. One ml aliquots of the plasma were transferred into separating funnels. Two mL of phosphate buffer (pH7.4) was added to each funnel. The contents were mixed, then extracted with 3 × 5 mL of chloroform. The organic layer was passed over anhydrous sodium sulphate and evaporated till dryness. The residue was dissolved in 10 mL of methanol. The recommended procedure under “Calibration Curve” was performed. A blank experiment was carried out simultaneously. Determine the nominal content of CLZ and IP using the following equation (18):

Recoveryin vivo=Deliveryin vivo×Recoveryin vitro/Deliveryin vitro

This means that % recovery for CLZ or IP in real human plasma=Concentration of the drug in real plasma X % recovery in spiked plasma/Concentration of the drug in spiked plasma.

## RESULTS AND DISCUSSION

### SDSF spectra of CLZ and IP

**Figure 2:**
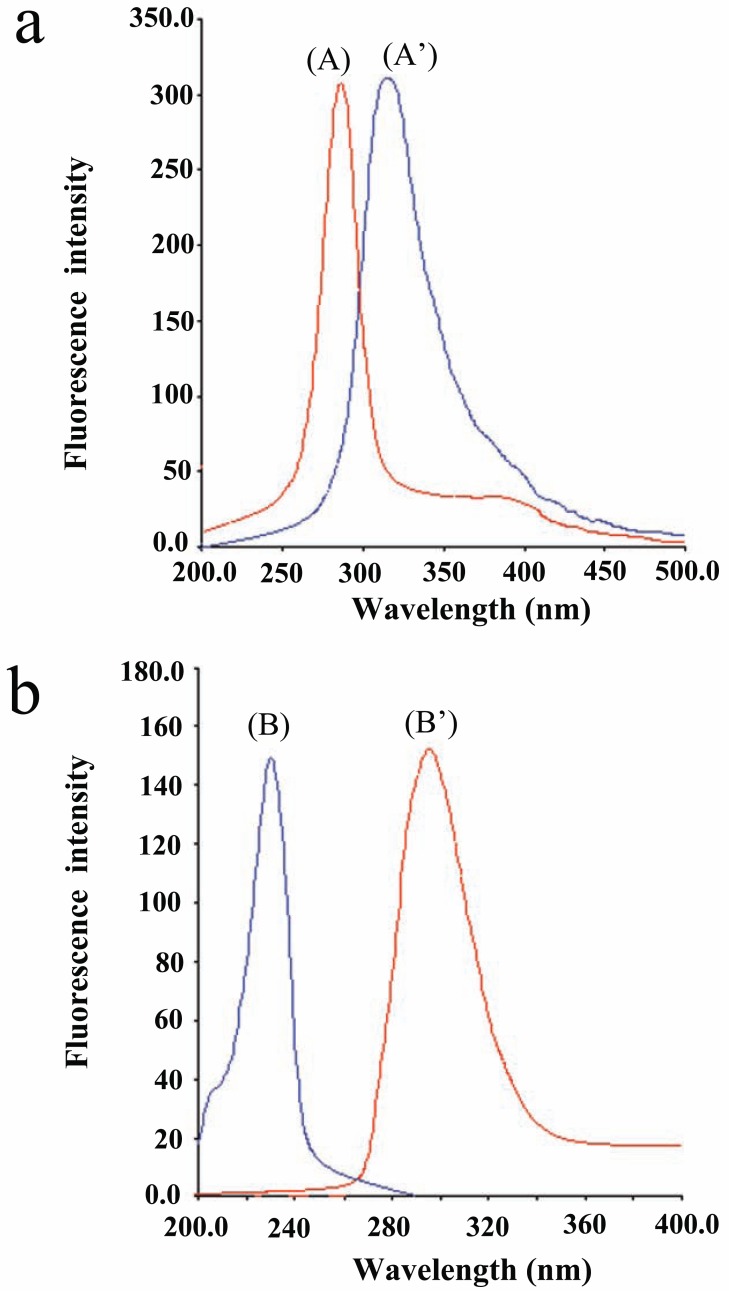
Normal fluorescence spectra at pH 7.0. a, (A, A’) are excitation and emission spectra of CLZ (1.0 μg/mL); b, (B, B’) are excitation and emission spectra of IP (0.8 μg/mL).

Both CLZ and IP exhibit native fluorescence with λ maximum of 312 nm and 295 nm, after excitation at 285 nm for CLZ and 231 for IP respectively (Fig. 2). Both the excitation and emission spectra of CLZ and IP overlapped (Fig. 2). This fact hindered the use of this method for the simultaneous determination of CLZ and IP. This problem is aggravated if it is desired to determine these compounds in their co-formulated preparations and biological fluids.

**Figure 3:**
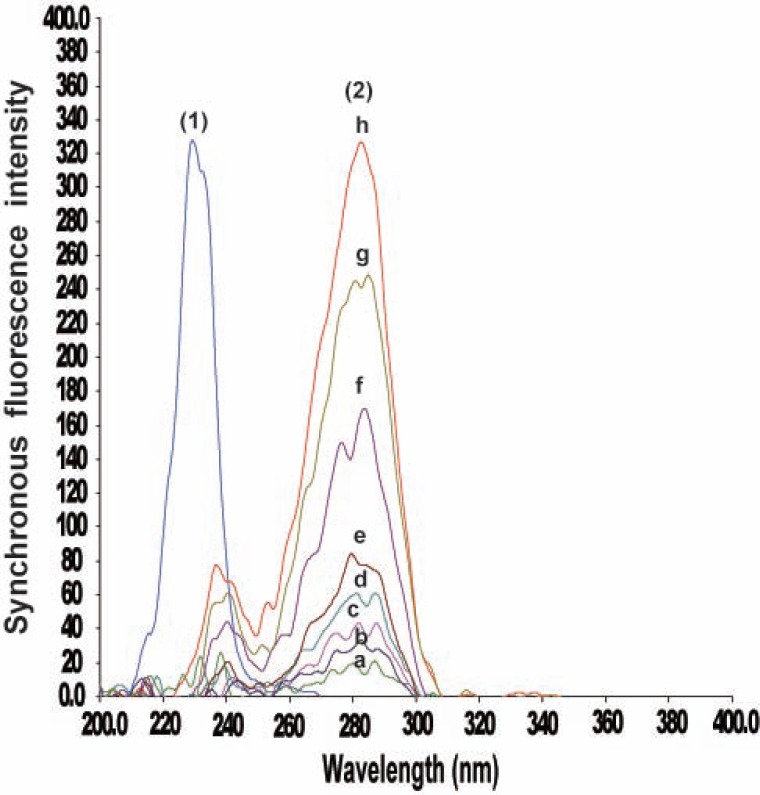
Synchronous fluorescence spectra of CLZ at 283 nm and IP. (1), spectrum of IP (0.6 μg/ mL); (2), a-h spectra of CLZ (0.2, 0.4, 0.5, 0.8, 1, 2, 3 and 4 μg/ mL) respectively.

**Figure 4:**
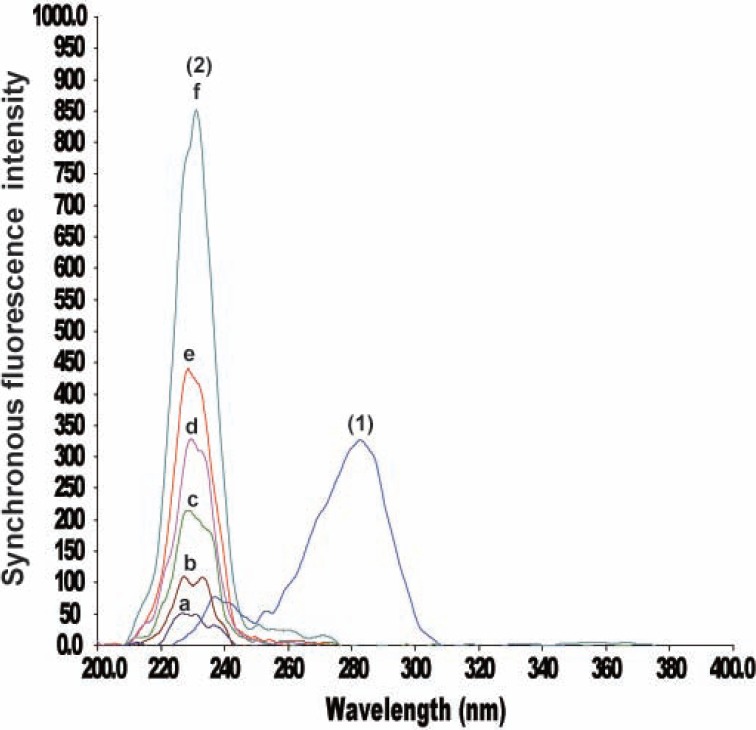
Synchronous fluorescence spectra of IP at 230 nm and CLZ. (1) Spectrum of CLZ (4.0 μg/mL); (2) a-f Spectra of IP (0.1, 0.2, 0.4, 0.6, 0.8 and 1.6 μg/mL) respectively.

It was necessary to first record, the normal synchronous spectra for CLZ and IP in order to derive the second derivative synchronous spectra. Fig. 3 shows the SF spectra of different concentrations of CLZ at 283 nm in presence of constant concentration IP (0.6 μg/mL), whereas, Fig. 4 illustrates the SF spectra of different concentrations of IP at 230 nm in presence of constant concentration of CLZ (4.0 μg/mL).

**Figure 5:**
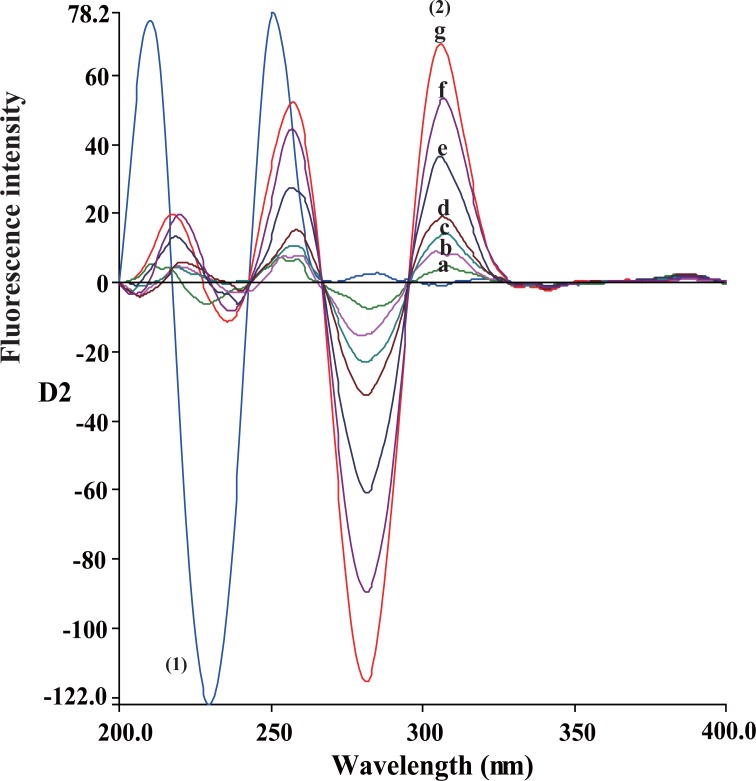
Second derivative synchronous fluorescence spectra of CLZ at 306 nm and IP. (1), Spectrum of IP (0.6 μg/mL); (2), a-g Spectra of CLZ (0.2, 0.5, 0. 8, 1, 2, 3 and 4.0 μg/mL) respectively.

**Figure 6:**
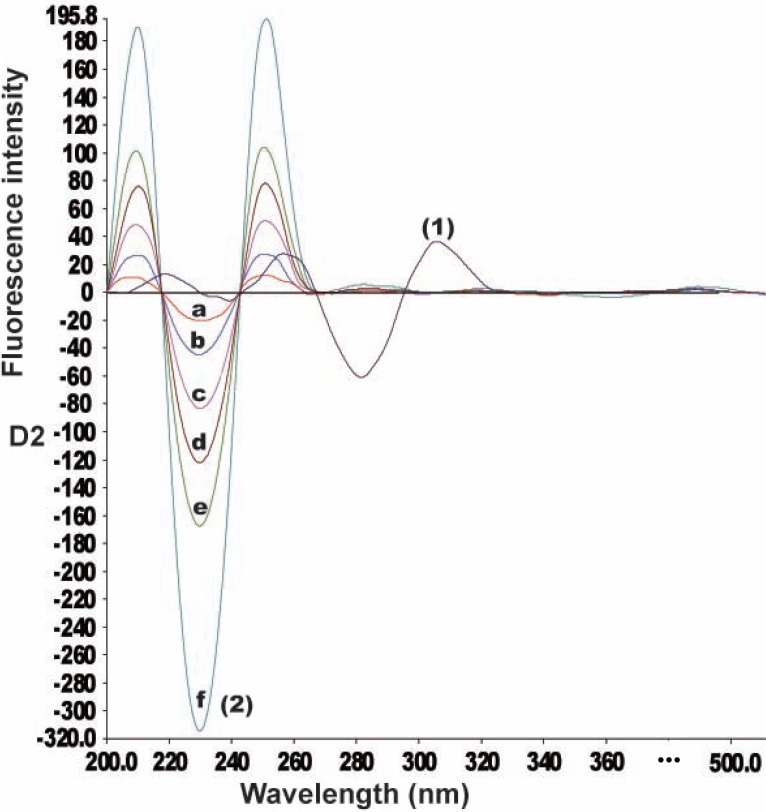
Second derivative synchronous fluorescence spectra of IP at 230 nm and CLZ. (1), Spectrum of CLZ (2.0 μg/mL); (2), a-f Spectra of IP (0.1, 0.2, 0.4, 0.6, 0.8 and 1.6 μg/mL) respectively.

Therefore we performed the SDSFS technique for simultaneous determination of each of CLZ and IP in their capsules. Spectra of CLZ and IP were well separated using SDSFS with a zero-crossing technique of measurement (Figs. 5 and 6). Under the experimental conditions the two peaks appeared at 306 and 230 nm for CLZ and IP respectively.

### Optimization of Reaction condition

Different experimental parameters affecting the performance of the proposed method were carefully studied and optimized. Such factors were changed individually while others were kept constant. These factors included Δλ selection, pH, type of the diluting solvent, stability time and ionic strength.

### Selection of optimum Δλ

The optimum Δλ value is important for performing the synchronous fluorescence scanning technique with regard to its resolution, sensitivity and features. It can directly influence spectral shape, band width and signal value. For this reason a wide range of Δλ (20, 40, 60, 80, 100 and 120 nm) was examined. When Δλ was less than 60 nm, the spectra shape were irregular, noisy and the fluorescence intensity is very weak. When Δλ was more than 60 nm, overlapping of the two peaks with poor separation was achieved. Therefore, Δλ of 60 was chosen as optimal for separation of CLZ and IP mixtures, since it resulted in two distinct peaks with good regular shapes and reduced the spectral interference caused by each compound.

### Effect of pH

**Figure 7:**
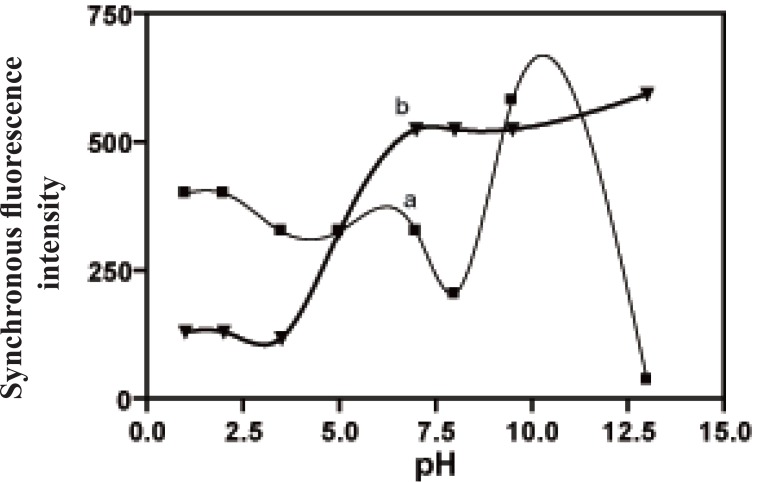
Effect of pH on the synchronous fluorescence intensity. a, ▪ CLZ (4.0 μg/mL) at 283 nm; b, ▲ IP (1.0 μg/mL) at 230 nm.

The influence of pH on the fluorescence intensity of the studied drugs was studied using different buffers covering the whole pH range, e.g. acetate buffer over the pH range of 3.6-5.6 and borate buffer over the pH range 6-9.5. The synchronous fluorescence intensity of CLZ remained constant with the increase of pH from 3.6 up to pH 7. Fluorescence intensity decreased at pH7.5, then it increased at pH 10.0, after which it decreased gradually up to pH 13 (Fig. 7). As for IP, increasing the pH values resulted in a gradual increase in the synchronous fluorescence intensity up to 7.0, then remained constant up to pH 9.50, after which it slightly increased at pH 13 (Fig. 7). Therefore, borate buffer of pH 7.0 was used throughout the study.

### Effect of volume of buffer

Increasing volume of borate buffer (pH 7.0) resulted in a gradual increase in the synchronous fluorescence intensity of CLZ and IP up to 2 mL after which the fluorescence intensity remained constant till 4 mL. Therefore, 2mL of borate buffer (pH 7.0) was chosen as the optimum buffer volume.

### Effect of diluting solvent

Dilution with different solvents including water, methanol, isopropanol, dimethyl sulfoxide (DMSO), tetrahydrofurane (THF) and dimethyl formamide (DMF) was employed. Of the all tested solvents methanol gave the heighest synchronous fluorescence intensities compared with the other solvents. Thus, methanol was chosen as the diluting solvent through out the study.

### Effect of time

The effect of time on the stability of the synchronous fluorescence intensity of the drugs was also studied. It was found that the fluorescence intensity developed instanteously and remained stable for more than 2 hours.

### Analytical Performance

The second derivative synchronous fluorescence spectroscopy-concentration plots for the two drugs were linear over the concentration range cited in Table 1. Linear regression analysis of the data gave the following equations:

D^2^=-0.16 + 17.50 C       (r=0.9999)

  for CLZ at 306 nm With S_a_=0.15 and S_b_=0.069

D^2^=0.846 + 206.0 C       (r=0.9999)

  for IP at 230 nm With S_a_=0.52 and S_b_=0.659

where D^2^ is the second derivative synchronous fluorescence spectroscopy, C is the concentration of the drug (μg/mL) and r is correlation coefficient.

The limit of quantification (LOQ) was determined by establishing the lowest concentration that can be measured according to ICH Q2B recommendations (19), below which the calibration graph is non linear. The limit of detection (LOD) was determined by evaluating the lowest concentration of the analyte that can be readily detected. The results of LOD and LOQ of CLZ and IP by SDSFS method are abridged in Table 1.

**Table 1:** Performance data of the proposed method

Parameter	Chlorzoxazone	Ibuprofen

Concentration range (μg/mL)	0.2–4.0	0.1–1.6
Correlation coefficient	0.9998	0.9999
Slope	17.50	206.0
Intercept	-0.106	0.846
Limit of detection (LOD) (μg/mL)	0.028	8.3 × 10^-3^
Limit of Quantification (LOQ) (μg/mL)	0.086	0.03
S_y/x_	0.243	0.52
S_a_	0.15	0.52
S_b_	0.069	0.659
%RSD	1.06	1.16
%Er	0.4	0.473

S_y/x_, Standard deviation of the residuals; S_a_, Standard deviation of the intercept % RSD=Relative standard deviation; S_b_, Standard deviation of the slope % Error=%RSD/√n.

LOQ and LOD were calculated according to ICHQ2 recommendations (19):

LOQ=10 σ/S

LOD=3.3 σ/S

S is the slope and σ is the standard deviation of the intercept of regression line of the calibration curve.

The proposed method was evaluated by studying the accuracy as percent relative error and precision as percent relative standard deviation. The results are abridged in Table 1.

Statistical analysis (20) of the results, obtained by the proposed and the official or reference methods (3, 5) using Student’s t-test and variance ratio F-test, shows no significant difference between the performance of the two methods regarding the accuracy and precision, respectively (Table 2). The reference method is based on the host-guest complexation between β-cyclodextrin (β-CD) and ibuprofen. The fluorescence of the resulted complex was measured at 290 nm after excitation at 230 nm (5).

**Table 2:** Application of the second derivative synchronous fluorimetry to the determination of the studied drugs in the pure form

Parameters	Concentration taken (μg/mL)	Concentration found (μg/mL)	% Found	Official or Reference methods (3, 5)

**1-CLZ**	0.2	0.2015	100.75	98.30
	0.5	0.4919	98.38	100.53
	0.8	0.7891	98.64	100.86
	1.0	1.008	100.80	99.43
	2.0	2.018	100.90	
	3.0	2.981	99.37	
	4.0	4.006	100.15	
X`			99.86	99.78
SD			1.06	1.16
t-test		0.116 (2.26)		
F value		1.2 (4.76)		
**2-IP**	0.1	0.0988	98.80	98.65
	0.2	0.2024	101.20	100.01
	0.4	0.3986	99.65	99.74
	0.6	0.5970	99.50	100.17
	0.8	0.8081	101.01	
	1.6	1.5730	98.32	
X`			99.75	99.64
SD			1.16	0.69
t-test		0.169 (2.31)		
F value		2.84 (5.41)		

Figures between parenthesis are the tabulated t and F values, respectively at p=0.05 (20).

### Analysis of synthetic mixture of CLZ and IP

**Figure 8:**
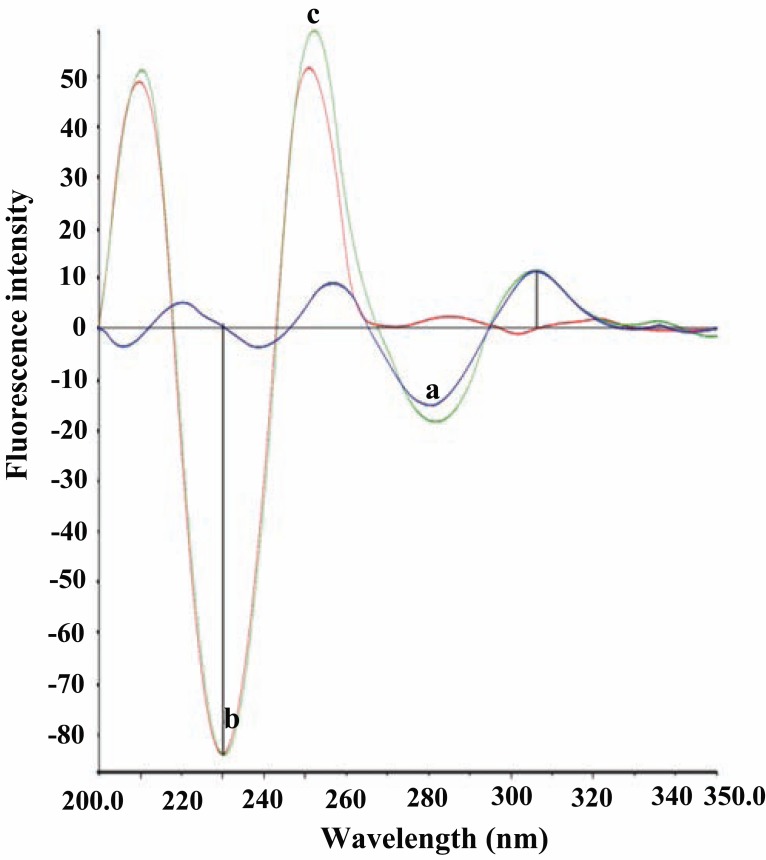
Second derivative synchronous fluorescence spectra. a, CLZ (0.5 μg/mL); b, IP (0.4 μg/mL); c, synthetic mixture of CLZ (0.5 μg/mL) and IP (0.4 μg/mL).

The proposed method was applied to the simultaneous determination of CLZ with IP in synthetic mixtures containing different concentrations of both drugs in a ratio of 5:4 (Fig. 8). The relative fluorescence intensities of second derivative technique were measured for both drugs. The second derivative signal of CLZ was measured at 306 nm which is considered as zero crossing point for IP and the second derivative signal for IP was measured at 230 nm which is the zero crossing point for CLZ. The concentrations of both drugs in the synthetic mixture were calculated according to the linear regression equation of the calibration graphs. The results indicate high accuracy of the proposed method as shown in Table 3.

**Table 3:** Application of the proposed method for determination of the studied drugs in their synthetic mixtures

Sample	Concentration taken (μg/mL)		Concentration found (μg/mL)		Recovery %	
	CLZ	IP	CLZ	IP	CLZ	IP

CLZ and IP mixture	0.25	0.2	0.2529	0.2014	101.16	100.70
	0.50	0.4	0.5088	0.4015	101.76	100.38
	0.75	0.6	0.7490		99.87	100.23
	1.0	0.8	1.0124	0.7873	101.24	98.41
X`					100.47	99.93
± SD					± 1.03	± 1.03
% RSD					1.03	1.03
% Error					0.515	0.515

Each result is the average of three separate determinations.

### Validation of the Method

The validity of the method was tested regarding; linearity, specificity, accuracy, repeatability and precision according to ICH Q2B recommendations. (19).

### Linearity

The regression plots showed a linear dependence of D^2^ values on drug concentration over the range cited in Table 1.

### Accuracy

The proposed methods were applied to the determination of authentic sample of CLZ and IP over the concentration range cited in Table 2 in order to determine their accuracy. The results obtained were in good agreement with those obtained using official (3) and reference methods (5). Using the Student’s t-test and the variance ratio F-test, (20) revealed no significant difference between the performance of the two methods regarding the accuracy and precision, respectively (Table 2).

The validity of the methods was proved by statistical evaluation of the regression lines, using the standard deviation of the residuals (S_y/x_), the standard deviation of the intercept (S_a_) and standard deviation of the slope (S_b_). The results are abridged in Table 1. The small values of the figures point out to the low scattering of the points around the calibration line and high precision.

### Precision

**Repeatability.** The repeatability was performed by applying the proposed methods for the determination of two concentrations of CLZ and IP in pure forms on three successive times, and the results are listed in Table 4.

**Table 4:** Validation of the proposed method for determination of CLZ and IP raw materials using SDSF mode

Concentration added (μg/ml)	% Recovery	% RSD	% Error

**CLZ**			
Intra-day			
1.0	100.31 ± 0.53	0.53	0.31
2.0	99.82 ± 0.70	0.70	0.40
Inter-day			
1.0	99.92 ± 0.80	0.80	0.46
2.0	100.15 ± 0.76	0.76	0.44
**IP**			
Intra-day			
0.80	100.72 ± 0.24	0.24	0.14
1.60	100.63 ± 0.53	0.53	0.31
Inter-day			
0.80	100.72 ± 0.79	0.79	0.46
1.60	100.62 ± 0.78	0.78	0.45

Each result is the average of three separate determinations.

**Intermediate precision.** Intermediate precision was evaluated through repeated analysis of CLZ and IP in pure form applying the proposed method, using the concentrations showed in Table 4, for a period of 3 successive days.

### Robustness of the method

The robustness of the proposed method is demonstrated by the constancy of the fluorescence intensity with the deliberated changes in the experimental parameters such as pH, 5 ± 1 for CLZ and pH, 8 ± 1 for IP, change volume of methanol and volume of buffer 3 ml ± 1. This minor change that may take place during the experimental operation didn’t greatly affect the fluorescence intensity of the mixture.

### Pharmaceutical Applications

The proposed method was applied to the determination of the studied drugs in their coformulated capsules. The specificity of the method was investigated by observing any interference encountered from the common excepients, such as lactose, gelatin, magnesium stearate and starch. These excepients did not interfere with the proposed method (Table 5).

**Table 5:** Application of the proposed method for determination of the studied drugs in their co-formulated preparations

Preparation	Concentration taken (μg/mL)		Concentration found (μg/mL)		Recovery %	
	CLZ	IP	CLZ	IP	CLZ	IP

Myofen capsules^a^	0.25	0.20	0.2497	0.2026	99.88	101.30
(CLZ 250 mg + IP 200 mg/capsule)	0.50	0.40	0.4928	0.3936	98.56	98.40
Batch # 604346	0.75	0.60	0.7569	0.5950	100.92	99.17
(x)					99.79	99.62
± SD					± 1.18	± 1.50
% RSD					1.18	1.50
% Error					0.68	0.87

Each result is the average of three separate determinations.

a Product of Eva Pharma for Pharmaceuticals and Medical Appliances S.A.E. Egypt.

### Biological applications

The high sensitivity of the proposed method allowed the determination of CLZ and IP in biological fluids by SDSFS method. The method was further applied to the *in-vivo* determination of both drugs in real human plasma.

IP is absorbed from gastro-intestinal tract. Following oral ingestion of a single oral dose of 250 mg CLZ and 200 mg IP, IP give a mean peak plasma 20-30 mg/L attained in about 1.1 hour (21). This value lies above the working concentration range of the proposed method. Thus they could be determined by the proposed method. The method involved extraction with chloroform. The extraction procedure described by Glowka *et al* (22) was adopted.

The within-day precision was evaluated through replicate analysis of real plasma containing CLZ and IP. The mean percentage recoveries based on the average of three separate determinations were 87.69 ± 6.15 and 92.57 ± 4.39 for CLZ and IP respectively. The results are abridged in Table 6.

**Table 6:** Application of the proposed method for determination CLZ and IP in real human plasma

Sample	Amount taken (μg/mL)		Amount found (μg/mL)		% Found	
	CLZ	IP	CLZ	IP	CLZ	IP

Real Plasma	0.25	0.20	0.2182	0.1788	87.28	89.40
	0.50	0.40	0.4702	0.3629	94.04	90.73
	1.0	0.80	0.8176	0.7806	81.76	97.58
(x)					87.69	92.57
± SD					± 6.15	± 4.39
% RSD					6.15	4.39
% Error					3.55	2.53

## CONCLUSION

A new simple and sensitive method was explored for the simultaneous determination of CLZ and IP in binary mixture. The second derivative synchronous spectrofluorometric method, by virtue of its high sensitivity, could be applied to the analysis of both drugs in their co-formulated dosage forms and biological fluids; it was possible to measure as concentrations low as 0.086 and 0.03 μg/mL for CLZ and IP respectively with good accuracy. Moreover, second derivative spectrofluorimetric technique enables the determination of CLZ in the presence of IP by applying the zero-crossing technique in the spectra without prior separation steps. Moreover, the proposed method is time saving.
